# Effect of first pass reperfusion on outcome in patients with posterior circulation ischemic stroke

**DOI:** 10.1136/neurintsurg-2021-017507

**Published:** 2021-05-04

**Authors:** Sanne J den Hartog, Bob Roozenbeek, Nikki Boodt, Agnetha A E Bruggeman, Adriaan C G M van Es, Bart J Emmer, Charles B L M Majoie, Ido R van den Wijngaard, Pieter Jan van Doormaal, Wim H van Zwam, Hester F Lingsma, Diederik W J Dippel, MR CLEAN Registry investigators, Diederik Dippel

**Affiliations:** 1 Neurology, Erasmus MC, University Medical Center, Rotterdam, The Netherlands; 2 Radiology and Nuclear Medicine, Erasmus MC, University Medical Center, Rotterdam, The Netherlands; 3 Public Health, Erasmus MC, University Medical Center, Rotterdam, The Netherlands; 4 Radiology and Nuclear Medicine, Amsterdam UMC Location AMC, Amsterdam, The Netherlands; 5 Radiology and Nuclear Medicine, Leiden University Medical Center, Leiden, The Netherlands; 6 Neurology, Haaglanden Medical Center, Den Haag, The Netherlands; 7 Neurology, Leiden University Medical Center, Leiden, The Netherlands; 8 Radiology and Nuclear Medicine, Maastricht University Medical Centre+, Maastricht, The Netherlands

**Keywords:** stroke, thrombectomy, brain, intervention

## Abstract

**Background:**

First pass reperfusion (FPR), that is, excellent reperfusion (expanded treatment in cerebral ischemia (eTICI) 2C-3) in one pass, after endovascular treatment (EVT) of an occluded artery in the anterior circulation, is associated with favorable clinical outcome, even when compared with multiple pass excellent reperfusion (MPR). In patients with posterior circulation ischemic stroke (PCS), the same association is expected, but currently unknown. We aimed to assess characteristics associated with FPR and the influence of FPR versus MPR on outcomes in patients with PCS.

**Methods:**

We used data from the MR CLEAN Registry, a prospective observational study. The effect of FPR on 24-hour National Institutes of Health Stroke Scale (NIHSS) score, as percentage reduction, and on modified Rankin Scale (mRS) scores at 3 months, was tested with linear and ordinal logistic regression models.

**Results:**

Of 224 patients with PCS, 45 patients had FPR, 47 had MPR, and 90 had no excellent reperfusion (eTICI <2C). We did not find an association between any of the patient, imaging, or treatment characteristics and FPR. FPR was associated with better NIHSS (−45% (95% CI: −65% to −12%)) and better mRS scores (adjusted common odds ratio (acOR): 2.16 (95% CI: 1.23 to 3.79)) compared with no FPR. Outcomes after FPR were also more favorable compared with MPR, but the effect was smaller and not statistically significant (NIHSS: −14% (95% CI: −51% to 49%), mRS acOR: 1.50 (95% CI: 0.75 to 3.00)).

**Conclusions:**

FPR in patients with PCS is associated with favorable clinical outcome in comparison with no FPR. In comparison with MPR, the effect of FPR was no longer statistically significant. Nevertheless, our data support the notion that FPR should be the treatment target to pursue in every patient treated with EVT.

## Introduction

Excellent reperfusion (expanded treatment in cerebral ischemia (eTICI) 2C-3) in one pass, first pass reperfusion (FPR), after endovascular treatment (EVT) of an occluded artery in the anterior circulation, is associated with favorable clinical outcome.^1 2^ In comparison with patients with anterior circulation ischemic stroke, patients with posterior circulation ischemic stroke (PCS) have not been studied extensively. Variables associated with outcome and reperfusion in patients with PCS are heterogeneous.^3^ It has been suggested that patients with PCS may have a different underlying pathophysiology than patients with anterior circulation ischemic stroke, which secondarily influences reperfusion and outcomes.^3^ Previous studies showed that in patients with a basilar artery occlusion, successful reperfusion is a strong predictor of favorable 90-day outcome.^4–8^ However, other studies showed more heterogeneity of clinical outcome despite high reperfusion rates.^9–11^

No studies have been published about the association between FPR and outcomes in patients with PCS. In patients with anterior circulation ischemic stroke, FPR is associated with favorable outcome, independently of patient, imaging, and treatment characteristics, even when compared with multiple pass excellent reperfusion (MPR).^1 12^ In patients with PCS, one might expect the same association, but this is currently unknown. Knowledge about the association of FPR with favorable clinical outcome in patients with PCS is needed to use FPR as a benchmark of good quality PCS care. We aimed to assess characteristics associated with FPR and assess the influence of FPR compared with MPR on clinical outcome in patients with PCS.

## Methods

We used data from the Multicenter Randomized Clinical Trial of Endovascular Treatment for Acute Ischemic Stroke in the Netherlands (MR CLEAN) Registry. This was a prospective observational study in all 18 centers performing EVT in the Netherlands. All patients undergoing EVT for acute ischemic stroke in the anterior and posterior circulation were registered, except for those who were treated in the BASICS trial.^13^ Detailed study design and methods have been described previously.^14 15^

### Patients

From the MR CLEAN Registry, we included patients aged 18 years or older, who had a symptomatic occlusion of the vertebral, basilar, or posterior cerebral artery confirmed by baseline computed tomography angiography. These data concerned patients who were treated with EVT between March 16, 2014 and December 31, 2018.

### Definition of FPR, clinical, imaging, and treatment characteristics

An imaging core laboratory analyzed all patient imaging. The members of the core laboratory were blinded to all clinical data. Reperfusion grade was measured according to the eTICI scale on final digital subtraction angiography (DSA) by the core laboratory. The number of attempts used to achieve reperfusion was based on the information given by the local treating interventionalist.

FPR was defined as a single pass of the device, without rescue treatment with intra-arterial thrombolytics, resulting in complete or near-complete reperfusion of the large vessel occlusion and its downstream territory: eTICI 2C-3. MPR was defined as eTICI 2C-3 after more than one pass or after one pass followed by rescue treatment with intra-arterial thrombolytics. No excellent reperfusion (NER) was defined as eTICI <2C independently of the number of passes.

Patient characteristics included: age, sex, history of atrial fibrillation, history of hypertension, history of diabetes mellitus, history of myocardial infarction, history of peripheral artery disease, history of stroke, history of hyperlipidemia, smoking, use of antiplatelet agents, use of vitamin K antagonists, use of direct oral anticoagulants, National Institutes of Health Stroke Scale (NIHSS) score at baseline, and pre-stroke modified Rankin Scale (mRS) score.

Imaging characteristics (scored by the core laboratory) included: level of obstruction, hyperdense artery sign, posterior circulation collateral score,^16^ posterior circulation Alberta Stroke Program Early CT Score (pc-ASPECTS),^17^ and vertebral artery dissection.

Treatment characteristics included: estimated time of large vessel occlusion (eLVO) to presentation at intervention hospital, presentation at intervention hospital to groin time, intravenous alteplase treatment, general anesthesia, and used device.

In patients with transient or mild neurological symptoms with secondary worsening consistent with the large vessel occlusion, the moment of secondary worsening was considered as the eLVO.^15^

### Outcomes

The percentage change in 24-hour NIHSS (±12 hours) was used as the primary outcome. This has been shown to be more closely related to EVT and reperfusion than the mRS score at 3 months, and has a good predictive value for long-term stroke outcome.^18^ We used the mRS scores at 3 months as a secondary outcome. Study staff were instructed to assess mRS scores at 90 days (±14 days).

### Missing data

All baseline data were reported as crude. If successful reperfusion was not achieved during EVT, we used the time of last contrast bolus injection as the final reperfusion time. For the use in regression models we imputed missing data using multiple imputation with R (package, MICE) based on relevant covariates and outcomes. Any mRS score of 0 to 5 at follow-up assessed within 30 days of symptom onset was considered invalid and treated as missing.

## Statistical analysis

We compared baseline characteristics of patients with FPR with patients without FPR using descriptive statistics. To investigate the association between patient, imaging, and treatment characteristics and FPR we used an univariable logistic regression model and selected all variables with a P value ≤0.2. These selected variables were used in a multivariable logistic regression model with a backward stepwise selection procedure with three steps. In each additional step, variables with a P value >0.2 were dropped, except for age and sex which were forced into the model. In step one, we tested all patient characteristics. In step two, we added all imaging characteristics to the remaining variables from step one. In step three, we added treatment characteristics to the remaining variables from step two. The final model consisted of all variables with a P value ≤0.2 and age and sex.

We analyzed the association between FPR and outcomes, adjusted for characteristics associated with FPR. First, we compared outcomes between FPR and no FPR. Second, we compared FPR with MPR. We used a linear regression model to analyze the NIHSS score at 24 hours and presented percentage change with 95% confidence intervals (CIs). Patients who had died before the time point of NIHSS assessment was reached, received the maximum NIHSS score of 42. The NIHSS was then log10 transformed, to better meet the assumption of normally distributed residuals in linear regression.^18^ We added one point to the NIHSS, so the original NIHSS of 0 was equivalent to log10 NIHSS +1. We used an ordinal logistic regression model to analyze the outcome mRS at 3 months and presented common odds ratios (ORs) with 95% CIs. We used the inverse of the mRS score for each patient. Lastly, we carried out a sensitivity analyses with a linear mixed model with random intercepts for hospitals and the primary outcome, NIHSS at 24 hours, to account for patient clustering within each hospital. All statistical analyses were performed with R statistical software (version 3.6.1).

## Results

**Figure 1:**
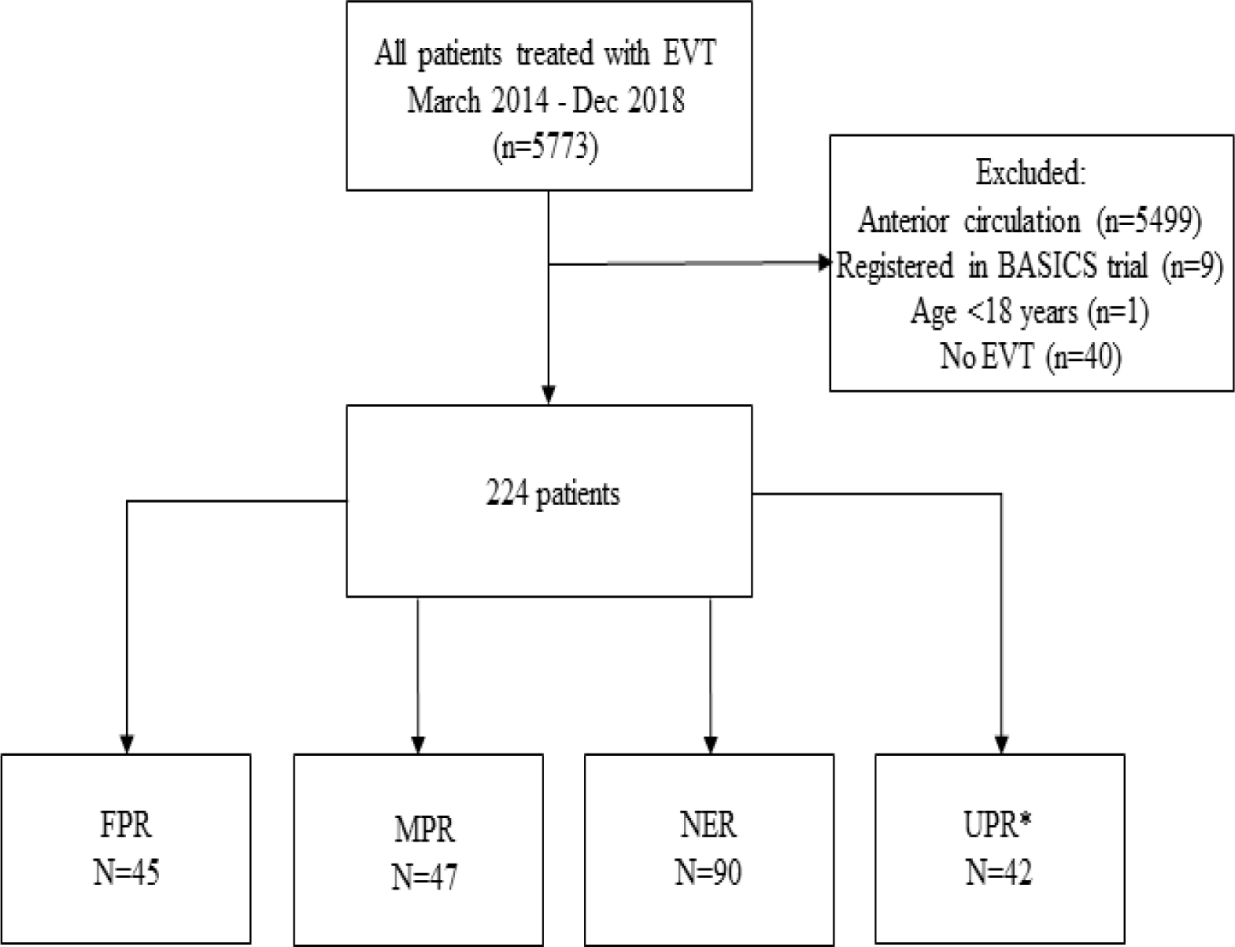
Flowchart of MR CLEAN Registry patients selected for analysis. *Thirty-four patients with a missing number of attempts, six patients with a missing eTICI score, and two patients with a missing number of attempts and missing eTICI score. eTICI, expanded treatment in cerebral ischemia; EVT, endovascular treatment; FPR, first pass reperfusion; MPR, multiple pass reperfusion;MR CLEAN, Multicenter Randomized Clinical Trial of Endovascular Treatment of Acute Ischemic Stroke in the Netherlands; NER, no excellent reperfusion; UPR, unclassified pass reperfusion.

Of all the patients in the MR CLEAN Registry, 264 adult patients had PCS. We excluded 40 patients who did not receive mechanical thrombectomy, because arterial access to the intracranial vasculature was not achieved or first DSA before EVT showed (spontaneous) reperfusion. Therefore, 224 patients were included (figure 1). FPR was achieved in 45/224 (20%) patients, MPR in 47/224 (21%), and NER in 90/224 (40%) patients. In 42 patients (19%) there was a missing number of attempts or missing eTICI score. These patients were allocated to the unclassified reperfusion (UPR) group. Baseline characteristics of the FPR, MPR, NER, and UPR group are shown in table 1.

**Table 1:** Baseline characteristics of patients with first pass reperfusion, multiple pass reperfusion, no excellent reperfusion, and unclassified reperfusion

Characteristic	FPR (n=45)	MPR (n=47)	NER (n=90)	UPR (n=42)
Age (years)	66 (60–73), 45	62 (53–76), 47	66 (54–74), 90	65 (52–77), 42
Women	53% (24/45)	40% (19/47)	39% (35/90)	50% (21/42)
Atrial fibrillation	27% (12/45)	15% (7/46)	11% (10/90)	10% (4/40)
Hypertension	51% (23/45)	52% (24/46)	51% (44/86)	46% (19/41)
Diabetes mellitus	9% (4/45)	11% (5/46)	18% (16/90)	24% (10/41)
Myocardial infarction	20% (9/45)	11% (5/45)	10% (9/88)	10% (4/41)
Peripheral artery disease	9% (4/44)	7% (3/45)	7% (6/88)	7% (3/41)
Previous ischemic stroke	22% (10/45)	11% (5/46)	21% (19/89)	12% (5/41)
Hyperlipidemia	35% (15/43)	13% (6/46)	18% (16/87)	15% (6/39)
Antiplatelets agents	36% (16/45)	22% (10/46)	29% (25/87)	25% (10/40)
Vitamin K antagonists	11% (5/45)	7% (3/46)	8% (7/88)	3% (1/39)
Direct oral anticoagulants	2% (1/45)	7% (3/46)	1% (1/87)	3% (1/38)
Baseline NIHSS	18 (11–25), 44	14 (10–21), 47	18 (11–35), 89	12 (6–31), 41
Pre-stroke mRS				
0–2	91% (41/45)	93% (42/45)	86% (76/88)	90% (36/40)
≥3	9% (4/45)	7% (3/45)	14% (12/88)	10% (4/40)
Level of obstruction				
Vertebral artery alone	2% (1/45)	7% (3/45)	6% (5/89)	7% (3/41)
Basilar artery alone*	47% (21/45)	51% (23/45)	38% (34/89)	37% (15/41)
BA extending into PCA	40% (18/45)	33% (15/45)	38% (34/89)	41% (17/41)
PCA	11% (5/45)	9% (4/45)	18% (16/89)	15% (6/41)
Hyperdense artery sign	62% (28/45)	52% (23/44)	64% (58/90)	50% (20/40)
pc-Collateral score	7 (5–8), 45	7 (5–8), 46	7 (5–8), 87	6 (5–8), 41
pc-ASPECTS	10 (9–10), 45	10 (9–10), 45	10 (9–10), 90	10 (9–10), 41
Vertebral artery dissection	12% (5/43)	13% (6/46)	14% (12/88)	25% (10/40)
Onset eLVO to door† time (min)	79 (42–164), 40	67 (13–148), 36	71 (25–179), 75	65 (0–121), 35
Symptom onset-door† time (min)	175 (93–133)	233 (146–386)	184 (76–356)	150 (74–339)
Transfer from PSC	44% (20/45)	55% (26/47)	38% (34/90)	33% (14/42)
IVT	51% (23/45)	40% (19/47)	49% (44/90)	48% (20/42)
Door† to groin time (min)	70 (51–102), 39	61 (37–90), 37	87 (55–124), 78	91 (60–137), 35
Procedure time (min)	35 (26–50), 42	76 (49–99), 38	70 (51–105), 82	62 (40–94), 37
Door† to reperfusion time (min)	93 (63–128), 41	107 (68–162), 42	157 (108–206), 82	150 (75–199), 37
General anesthesia	56% (25/45)	60% (28/47)	54% (48/89)	55% (22/40)
First used device				
Stent retriever	60% (27/45)	57% (27/47)	68% (61/90)	59% (23/39)
Aspiration device	40% (18/45)	40% (19/47)	27% (24/90)	33% (13/39)
Other‡	0%	2% (1/47)	6% (5/90)	8% (3/39)
Post eTICI				
2C	18% (8/45)	26% (12/47)	0	12% (4/34)
3	82% (37/45)	75% (35/47)	0	56% (19/34)
Median number of attempts	1	3 (2–4), 47	2 (1–4), 90	–

Categorical variables are presented as percentage (n/N). Continuous variables are presented as median (IQR), N.

*In two patients there was no full obstruction.

†Door intervention center.

‡Balloon dilatation, stent.

BA, basilar artery; eLVO, estimated time of large vessel occlusion; eTICI, expanded thrombolysis in cerebral infarction; FPR, first pass reperfusion; IQR, interquartile range; IVT, intravenous alteplase treatment; MPR, multiple pass reperfusion; mRS, modified Rankin Scale; NER, no excellent reperfusion; NIHSS, National Institutes of Health Stroke Scale; pc, posterior circulation; PCA, posterior cerebral artery; pc-ASPECTS, posterior circulation Alberta Stroke Program Early CT Score; PSC, primary stroke center; UPR, unclassified reperfusion.

### Characteristics associated with FPR

We analyzed the association of patient, imaging, and treatment characteristics with FPR compared with no FPR (MPR+NER). In the backward stepwise selection procedure only a history of hyperlipidemia was selected, next to age and sex which were forced into the model. None of these variables were associated with FPR (history of hyperlipidemia adjusted odds ratio (aOR) 1.17 (95% CI: 0.99 to 1.39)). We also analyzed the association of patient, imaging, and treatment characteristics with FPR contrasted with MPR. We selected age, sex, history of hyperlipidemia, and baseline pc-ASPECTS from the backward stepwise selection procedure. However, none of these variables were associated with FPR (history of hyperlipidemia aOR 1.27 (95% CI: 0.98 to 1.63), pc-ASPECTS aOR 1.04 (95% CI: 0.95 to 1.13)).

### Association between FPR and NIHSS at 24 hours

In the univariable regression analysis, FPR compared with no FPR led to a statistically significant decrease in 24-hour NIHSS score of −45% (95% CI: −64 to −14) (table 2). Compared with MPR the reduction in 24-hour NIHSS score was −22% (95% CI: −54 to 32). In the multivariable analyses, we adjusted for characteristics selected from the backward stepwise selection procedure. FPR still led to a reduction in 24-hour NIHSS score compared with no FPR (−45% (95% CI: −65 to −12)) and compared with MPR (−14% (95% CI: −51 to 49)). We found no differences in the results when we adjusted for patient clustering within each hospital (online supplemental data table S1) (ICC=0.05).

10.1136/neurintsurg-2021-017507.supp1 Supplementary data

**Table 2:** Univariable and multivariable linear/ordinal logistic regression for the association between first pass reperfusion (FPR) and 24-hour National Institutes of Health Stroke Scale score and FPR and modified Rankin Scale score at 3 months

Comparison	NIHSS at 24 hours percentage change		mRS at 3 months	
	% (95% CI)	Adjusted % (95% CI)	cOR (95% CI)	acOR (95% CI)
FPR vs no FPR	−45%(−64 to −14)	−45%(−65 to −12)*	1.96 (1.14 to 3.39)	2.16 (1.23 to 3.79)*
FPR vs MPR	−22%(−54 to 32)	−14%(−51 to 49)†	1.33 (0.69 to 2.53)	1.50 (0.75 to 3.00)†

*Adjusted for age, sex, history of hyperlipidemia.

†Adjusted for age, sex, history of hyperlipidemia, posterior circulation Alberta Stroke Program Early CT Score (pc-ASPECTS).

acOR, adjusted common odds ratio; cOR, common odds ratio; eTICI, expanded treatment in cerebral ischemia; FPR, first pass reperfusion; MPR, multiple pass reperfusion; mRS, modified Rankin Scale,; NIHSS, National Institutes of Health Stroke Scale; no FPR, MPR (eTICI ≥2C in multiple passes) + NER (eTICI <2C, independent of number of passes).

Procedure time for FPR was shorter than for no FPR (table 1). When procedure time was added to the adjustments there was still a benefit of FPR compared with no FPR (−39% (95% CI: −63 to −2)) and compared with MPR (−6% (95% CI: −53 to 89)).

### Association between FPR and MRS score at 3 months

**Figure 2:**
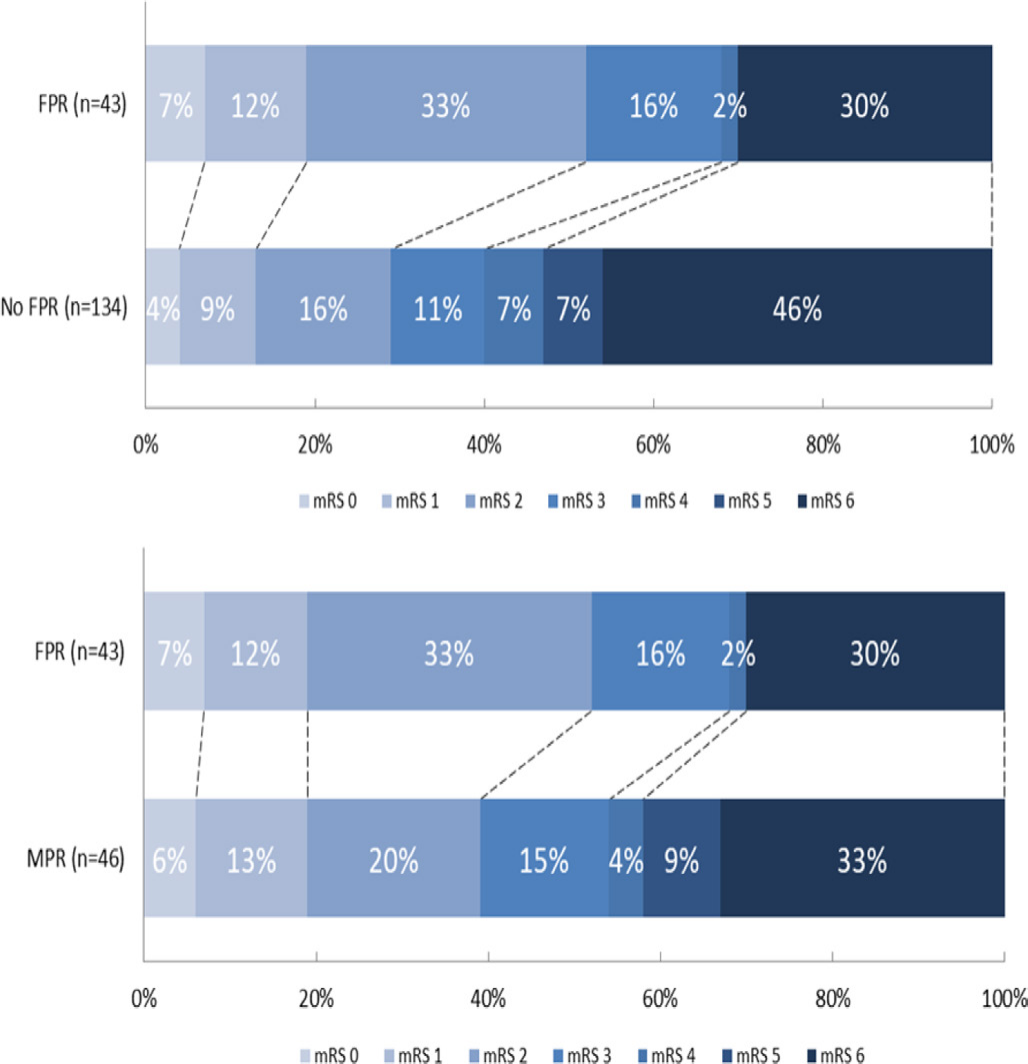
Modified Rankin Scale (mRS) scores at 3 months, first pass reperfusion (FPR) versus no FPR and FPR versus multiple pass reperfusion (non-imputed data). eTICI, expanded thrombolysis in cerebral infarction; FPR, first pass reperfusion; MPR, multiple pass reperfusion; no FPR, MPR (eTICI ≥2C in multiple passes) + no excellent reperfusion (NER) (eTICI <2C, independent of number of passes).

In the univariable regression analyses, FPR led to more favorable mRS scores at 3 months compared with no FPR (cOR 1.96 (95% CI: 1.14 to 3.39)) and compared with MPR (cOR 1.33 (95% CI: 0.69 to 2.53)) (table 2). The shift on the mRS scale is shown in figure 2. In the multivariable analysis, FPR led to more favorable mRS scores at 3 months compared with no FPR (acOR 2.16 (95% CI: 1.23 to 3.79)) and compared with MPR (acOR 1.50 (95% CI: 0.75 to 3.00)). When procedure time was added to the adjustments the effect of FPR over MPR was reduced (acOR 1.19 (95% CI: 0.56 to 2.55)).

## Discussion

In our study, FPR was associated with favorable clinical and functional outcomes. There were no patient, imaging, or treatment characteristics associated with FPR. In a study of patients with PCS treated with aspiration first, a history of diabetes, onset-to-groin time, and cardioembolic etiology were found as predictors of FPR (in this study defined as eTICI ≥2B in one attempt).^19^ We did not find such associations in our analysis, but no separate analysis for aspiration first and stent retriever thrombectomy were done. In two systematic reviews, aspiration as first attempt appeared to be superior to stent retriever in achieving modified treatment in cerebral infarction (mTICI) 2b-3 in patients with PCS,^3 20^ which is not in line with our results. However, in the ASTER trial where patients with an anterior circulation ischemic stroke were randomly assigned to EVT with a stent retriever or aspiration, similar rates of FPR were achieved in both groups.^21^ In patients with an anterior circulation ischemic stroke there is a strong association between location of the occluded artery and FPR.^1 22^ We did not find an association between FPR and the location of the occluded artery in the posterior circulation. However, most of the patients in our cohort had a basilar artery occlusion (177/224, 79%), whereas 31 patients (14%) had a posterior cerebral artery occlusion and only 12 patients (5%) had a vertebral artery occlusion. In the FPR group there was only one patient with a vertebral artery occlusion. We do not have an explanation for this low number, probably this is due to chance. Our study population is heterogeneous as regards the location of the occluded artery. The likelihood of a good outcome may differ between these localizations, but relative effects of intervention less so. We therefore do not think that location influenced our results.

The association between FPR and 24-hour NIHSS is comparable to our results in patients with anterior circulation ischemic stroke (compared with no FPR; −45% vs −37% compared with MPR −14% vs −23%).^12^ In contrast to patients with anterior circulation ischemic stroke, in patients with PCS there is no significant benefit of FPR over MPR in our results. In a previous study with a pooled analysis of patients with either anterior or posterior circulation stroke, no differences in outcome (dichotomized mRS) between the FPR and MPR group were noted.^19^ In a study with a small number of patients (FPR n=19 and MPR n=13) with a basilar artery occlusion there was also no difference in outcomes between the FPR and MPR group.^23^ There are, however, no studies describing the effect of FPR versus MPR on outcome in a larger sample of patients with only PCS. The differences in effect of FPR over MPR on outcome between patients with anterior circulation stroke and patients with PCS suggests a different pathophysiology, vascular anatomy, and outcomes in patients with PCS compared with patients with anterior circulation ischemic stroke.^3^ Although the effect of FPR over MPR is not significant as regards the 24-hour NIHSS and mRS score at 3 months, the observed percentage change in NIHSS and odds of a favorable mRS score is high. Therefore, we may conclude that FPR should be the treatment target to pursue in every patient treated with EVT.

The effect of FPR over MPR and NER can be partly explained by a shorter procedure time. We showed that the effect of FPR on outcomes was not completely reduced when we adjusted for procedure time. A higher maneuver count is associated with a poor outcome despite successful recanalization.^24^ Explanations for these poor outcomes could be an increase in complications, vessel wall damage, thrombus migration, and microembolization.^1 25 26^

### Limitations

Parallel to the data collection of the MR CLEAN Registry, the BASICS trial was performed. Of all the hospitals in the MR CLEAN Registry, 10 participated in the BASICS trial and eight did not participate in the BASICS trial. There could be some selection bias in our cohort because we only collected data of patients treated with EVT outside the BASICS trial. In separate analyses of our data there were no differences in patient characteristics and outcomes between patients treated in a BASICS trial center and patients treated in an non-BASICS trial center.^15^ Moreover, our cohort is similar as regards baseline characteristics and outcomes to other registries on EVT for patients with PCS.^5 15 27 28^

All our imaging was assessed by an independent core laboratory. We used very strict definitions for these assessments, and when the quality of imaging was low or there were only a few images available the imaging variables were scored as missing. Registries in general are prone to missing values. However, all data were verified by our study coordinators, and we made every effort to retrieve the missing data.^13^ To use the missing data in a responsible way and to avoid bias, the missing values were imputed by means of multiple imputation.

During the inclusion period of the MR CLEAN Registry there was no clear evidence for a benefit of EVT over best medical management on outcome in patients with a basilar artery occlusion.^29^ Therefore, the treatment of patients with PCS differed between hospitals. This could result in bias and a clustering effect. The sensitivity analyses with a linear mixed model, to account for this clustering, did not show any differences in outcome compared with the results of the linear regression model.

Another limitation is the assessment of the eTICI score, which is less reliable in the posterior circulation. The assessment is more difficult due to the interference with abundant collateral flows, incomplete visualization of the perforating arteries to the brain stem, and the necessity to consider the antegrade flow from the anterior circulation.^30^ Our core laboratory was well instructed to assess the eTICI score. However, a reliable eTICI score is fundamental for the definition of FPR. Further research is needed on how to assess the eTICI score in the posterior circulation.

## Conclusions

FPR in patients with PCS is associated with favorable clinical outcome in comparison with no FPR. In comparison with MPR, the effect of FPR was no longer statistically significant. Nevertheless, our data support the notion that FPR should be the treatment target to pursue in every patient treated with EVT.

## Data Availability

In compliance with the General Data Protection Regulation, source data are not available for other researchers. Information about analytic methods, study materials and scripts of the statistical analyses are available from the corresponding author on reasonable request.
