# Supplementary material for: Effect of first pass reperfusion on outcome in patients with posterior circulation ischemic stroke
Source: J Neurointerv Surg. 2021 May 4;14(4):333–40. doi: 10.1136/neurintsurg-2021-017507 (PMC8938660; doi:10.1136/neurintsurg-2021-017507)
Supplement: Supplementary data [file neurintsurg-2021-017507supp001.pdf]

SUPPLEMENTAL MATERIAL

The effect of first pass reperfusion on outcome in patients with posterior circulation ischemic stroke.

Supplement Table S1. Association between FPR and 24-hour NIHSS score with adjustment for patient clustering within each hospital.

| | NIHSS at 24 hours Percentage change | |
|---|---|---|
| | % (95%CI) | Adjusted % (95%CI) |
| FPR vs no FPR | -45% (-64 to -14) | -45% (-65 to -13)* |
| FPR vs MPR | -23% (-55 to 33) | -15% (-52 to 50)† |

NIHSS, National Institutes of Health Stroke Scale, FPR, first pass reperfusion, MPR multiple pass reperfusion,

no FPR = MPR (eTICI ≥2C in multiple passes) + no excellent reperfusion (eTICI <2C, independent of number of passes)

* adjusted for age, sex, history of hyperlipidemia.

† adjusted for age, sex, history of hyperlipidemia, posterior circulation Alberta stroke program early CT score.
